# Towards an understanding of salient neighborhood boundaries: adolescent reports of an easy walking distance and convenient driving distance

**DOI:** 10.1186/1479-5868-4-66

**Published:** 2007-12-18

**Authors:** Natalie Colabianchi, Marsha Dowda, Karin A Pfeiffer, Dwayne E Porter, Maria João CA Almeida, Russell R Pate

**Affiliations:** 1 Department of Epidemiology and Biostatistics, University of South Carolina, 921 Assembly Street, Columbia, SC 29208, USA; 2 Department of Exercise Science, University of South Carolina, 921 Assembly Street, Columbia, SC 29208, USA; 3 Department of Kinesiology, Michigan State University, Room 3 IM Sports Circle, East Lansing, MI 48824, USA; 4 Department of Environmental Health Sciences, University of South Carolina, 921 Assembly Street, Columbia, SC 29208, USA; 5 Department of Physical Education and Sports, University of Madeira, 9000-390 Funchal, Madeira, Portugal

## Abstract

Numerous studies have examined the association between the surrounding neighborhood environment and physical activity levels in adolescents. Many of these studies use a road network buffer or Euclidean distance buffer around an adolescent's home to represent the appropriate geographic area for study (i.e., neighborhood). However, little empirical research has examined the appropriate buffer size to use when defining this area and there is little consistency across published research as to the buffer size used. In this study, 909 12^th ^grade adolescent girls of diverse racial and geographic backgrounds were asked to report their perceptions of an easy walking distance and a convenient driving distance. These two criterions are often used as the basis for defining one's neighborhood.

The mean easy walking distance in minutes reported by adolescent girls was 14.8 minutes (SD = 8.7). The mean convenient driving distance in minutes reported was 17.9 minutes (SD = 10.8). Nested linear multivariate regression models found significant differences in reported 'easy walking distance' across race and BMI. White adolescents reported on average almost 2 minutes longer for an easy walking distance compared to African American adolescents. Adolescents who were not overweight or at risk for overweight reported almost 2 minutes fewer for an easy walking distance relative to those who were overweight or at risk for overweight. Significant differences by urban status were found in the reported 'convenient driving distance'. Those living in non-urban areas reported on average 3.2 minutes more driving time as convenient compared to those living in urban areas. Very little variability in reported walking and driving distances was explained by the predictors used in the models (i.e., age, race, BMI, physical activity levels, urban status and SES).

This study suggests the use of a 0.75 mile buffer to represent an older female adolescent's neighborhood, which can be accessed through walking. However, determining the appropriate area inclusive of car travel should be tailored to the geographic location of the adolescent since non-urban adolescents are willing to spend more time driving to destinations. Further research is needed to understand the substantial variability across adolescent perceptions of an easy walking and convenient driving distance.

## Background

There has been a dramatic increase in the number of studies that examine the association between characteristics of an adolescent's neighborhood and physical activity. While many studies have found positive associations [1,2], there is surprisingly little consistency across studies in the operational definition of one's neighborhood.

Earlier research used the census tract as the basis for describing a neighborhood [3]. More recently buffers around the participant's home are used as a proxy for a neighborhood [4]. Commonly used distances for creating these buffers in the adolescent physical activity literature are 0.5–0.6 miles (800 – 1000 meters) [5-7] or a 1 mile (1500–1600 meters) buffer around the home [8-10]. However buffers as small as 0.25 miles (400 meters) [11] and as large as 5 miles (8.05 kilometers) have been used [12]. Most studies acknowledge the lack of empirical data for selecting these distances and suggest additional research. One rationale for selecting these geographies is that they correspond to an easy walking distance [7,11] though little empirical evidence exists for defining an easy walking distance. Larger buffers are sometimes employed to represent a reasonable driving distance [13].

The few studies that have explicitly studied perceptions of neighborhood boundaries have been completed with adults [14,15]. However the perception of neighborhood boundaries for an adolescent would likely be different. Also notably absent from the literature are studies that examine differences in neighborhood boundaries across other characteristics, such as whether the person lives in an urban or rural area. This study addresses this gap by explicitly examining adolescent reports of an easy walking distance and convenient driving distance.

## Methods

This study includes female adolescents recruited as part of a physical activity intervention trial (N = 1655) [16], who also completed a physical activity recall survey, an addendum with walking and driving distance questions and whose address could be geocoded (59%). After participants with missing covariates were deleted, the final sample included 909 12^th ^grade adolescent girls. The sample contained nearly equal numbers of White and African American adolescents. Parental consent and adolescent assent/consent were obtained. The procedures were approved by the Institutional Review Board at the University of South Carolina.

The main outcomes in this study are reported 'easy walking distance' and 'convenient driving distance' in minutes. These outcomes were calculated from these two questions: "When you think of an 'easy walking distance', it is ____ minutes"; and "When you think of a 'convenient driving distance', it is ____ minutes". The distribution of these outcomes was skewed to the right and therefore all values above the 95^th ^percentile were recoded to the 95^th ^percentile.

The mean number of minutes was calculated for easy walking distance. Linear regressions, which controlled for school effects and intervention status, were completed using SAS™ version 9.1 with minutes of easy walking distance as the dependent variable and the following as independent variables: race, urban status, SES, BMI, physical activity level and age. The covariates were classified as follows: Race (White or African American); Urban status (based on participant's census block); SES (parent with ≤ high school versus > high school education); Objectively measured BMI (at risk for overweight or overweight defined as ≥ 85^th ^percentile using CDC growth charts versus <85^th ^percentile); Physical activity level (two or more 30-min blocks of moderate-to-vigorous physical activity per day self reported on the 3-Day Physical Activity Recall [17] versus less activity); and Age (years). Regressions were first run with each predictor variable examined separately while controlling for intervention status and school effects. Then linear regressions with all predictors examined simultaneously were run. All analyses were then repeated using 'convenient driving distance' as the outcome.

## Results

The mean easy walking distance reported was 14.8 minutes (SD: 8.7). In single predictor models, significant differences were found in reported 'easy walking distance' across race (β for white adolescents = 1.27; p = .03) and weight group (β for non-overweight/non-at risk for overweight = -1.65; p = .01). The multivariate analysis mirrored the single predictor models (see Table 1). White adolescents reported on average almost 2 minutes longer for an easy walking distance compared to African American adolescents. Adolescents who were not overweight or at risk for overweight reported almost 2 minutes fewer for an easy walking distance relative to those who were overweight or at risk for overweight. Age, urban status, SES and physical activity levels were not significantly associated with reported easy walking distance in these analyses.

**Table 1:** Regression analyses for the relationship between 'Easy Walking Distance' and 'Convenient Driving Distance' with demographic characteristics controlling for intervention status and with school as a random variable

	Easy Walking Distance		Convenient Driving Distance	
	**β (SE)**	**p-value**	**β (SE)**	**p-value**

Intercept	1.51 (8.44)	.86	17.26 (10.46)	.11
Group (Control)	0.63 (0.69)	.37	1.27 (0.72)	.09
Age	0.75 (0.47)	.11	-0.02 (0.59)	.97
Race (White)	1.83 (0.62)	.003	-1.23 (0.76)	.10
SES (Having a parent with HS education or less)	0.79 (0.62)	.20	0.88 (0.76)	.25
BMI (Not overweight or at risk for overweight)*	-1.86 (0.63)	.003	-0.86 (0.78)	.27
Physical Activity (Insufficient MVPA)**	-0.32 (0.66)	.63	-0.12 (0.82)	.88
Urban status (Non-urban)	0.07 (0.61)	.90	3.24 (0.73)	<.001

* < 85^th ^percentile using CDC growth charts for adolescents; <25 BMI for participants old enough to be classified as adults

** Zero or one 30-min blocks of physical activity at ≥ 3 METS

The mean convenient driving distance reported was 17.9 minutes (SD: 10.8). In single predictor models, significant differences were found in reported 'convenient driving distance' across race (β for white adolescents = -1.49; p = .04), SES (β for having a parent with high school education or less = 1.58; p = .04), and urban status (β for non-urban adolescents = 3.34; p < .001). Age, BMI and physical activity levels were not significantly associated with reported convenient driving distance in the single predictor models. In the multivariate model, only urban status remained significantly associated with reported convenient driving distance (see Table 1). Those living in non-urban areas reported on average 3.2 minutes more driving time as convenient compared to those living in urban areas.

There were large standard deviations for both easy walking distance and convenient driving distance. A model concordance correlation coefficient (MCCC) was calculated for the two models. The MCCC is similar to an R^2 ^in a linear regression but is appropriate for mixed models in that it accounts for non-independent responses [18]. The MCCC was 0.06 for both multivariate models indicating that very little variability in reported walking and driving distances was explained by the predictors in these models.

## Discussion

On average, female adolescents reported that an easy walking distance was about 15 minutes. Assuming a walking speed of 80 meters per minute or 3 miles per hour [19], a 15 minute walk translates to 1184 meters or approximately 0.75 miles. However there was substantial variation across adolescent reports that was not explained by the predictor variables included in the models. Consequently, while a 15 minute walk may be an easy distance for many older female adolescents, further research is needed to understand the great variability that remains across adolescents. The mean reported convenient driving distance was 17.9 minutes. Assuming an average driving speed of 55 kilometers per hour or 35 miles per hour, a 17.9 minute drive translates to 16.4 kilometers or approximately 10 miles.

Non-urban adolescents reported a longer distance as convenient for driving compared to urban adolescents but there was not a significant difference in the reported easy walking distance between urban and non-urban adolescents. This suggests that non-urban adolescents may be willing to drive farther however there may be a common limit to how far adolescents are willing to walk. The significant differences in an easy walking distance by race may be confounded with the distance needed to travel to get to destinations as recent research suggests that the distance adults are willing to walk may depend on the destination [20]. At present, it is not known why adolescents who were not overweight or at risk for overweight would report a shorter distance as 'easy' though it could be related to a quicker walking pace which would reduce the amount of time it would take to walk the same distance.

This study is the first to explicitly examine the distance that adolescents perceive as an easy walking distance and convenient driving distance. A study of parental reports of an appropriate walking distance for their children reported that 1.5 km (about 1 mile) for children 5–6 and 1.6 km for children 10–12 years of age were considered appropriate [21]. Other researchers have attempted to determine the appropriate buffer size by investigating the persistence of a relationship between physical activity and the built environment across various buffers [8,9,22] or have examined threshold distances [23]. These studies have generally supported buffers ranging from 0.25 to 1 mile.

Since this study examined only females in 12^th ^grade in one southeastern US state, further research should be completed to understand what constitutes an 'easy walking distance' and 'convenient driving distance' for younger adolescents, boys and adolescents in different locations. Further, adolescents were asked to report an *easy *walking distance and *convenient *driving distance though adolescents may need to or choose to walk further than an easy distance or drive further than a convenient distance for a variety of reasons. In addition, in translating the reported minutes of an easy walking distance and convenient driving distance into an actual distance, a constant walking rate and driving rate was assumed for all adolescents. Finally, the buffers described utilized the mean minutes reported. Shorter times were preferred by over half the sample and under half of participants reported longer times. Caution should be taken when assigning any single buffer across all adolescents as there is great variability across definitions.

## Conclusion

This study supports the postulation that 0.75 miles is an appropriate walking distance for older adolescent girls. The distance representing a convenient driving distance should be tailored to the geographic location of the adolescent since non-urban adolescents found longer driving times more convenient.

## Competing interests

The author(s) declare that they have no competing interests.

## Authors' contributions

NC, KP, MA, MD, DP and RP contributed to the conception and design of the study. RP originated the study and supervised all aspects of its implementation. MD served as data manager and completed the analyses. All authors were involved the interpretation of the data and the drafting and revision of content. All authors read and approved the final manuscript.
